# Stroke Prophylaxis in Patients with Atrial Fibrillation and End-Stage Renal Disease

**DOI:** 10.3390/jcm9010123

**Published:** 2020-01-02

**Authors:** Martin van Zyl, Hafez M. Abdullah, Peter A. Noseworthy, Konstantinos C. Siontis

**Affiliations:** 1 Department of Cardiovascular Medicine, Mayo Clinic, Rochester, MN 55905, USA; vanzyl.martin@mayo.edu (M.v.Z.); noseworthy.peter@mayo.edu (P.A.N.); 2 Department of Internal Medicine, University of South Dakota Sanford School of Medicine, Vermillion, SD 57069, USA; Ammar.Abdullah@usd.edu

**Keywords:** atrial fibrillation, end-stage renal disease, anticoagulation, stroke prevention

## Abstract

Atrial fibrillation (AF) is an important comorbidity in patients with end-stage renal disease (ESRD) undergoing dialysis that portends increased health care utilization, morbidity, and mortality in this already high-risk population. Patients with ESRD have a particularly high stroke risk, which is further compounded by AF. However, the role of anticoagulation for stroke prophylaxis in ESRD and AF is debated. The ESRD population presents a unique challenge because of the combination of elevated stroke and bleeding risks. Warfarin has been traditionally used in this population, but it is associated with significant risks of minor and major bleeding, particularly intracranial, thus leading many clinicians to forgo anticoagulation altogether. When anticoagulation is prescribed, rates of adherence and persistence are poor, leaving many patients untreated. The direct oral anticoagulants (DOACs) may offer an alternative to warfarin in ESRD patients, but these agents have not been extensively studied in this population and uncertainties regarding comparative effectiveness (versus warfarin, each other, and no treatment) remain. In this review, we discuss the current evidence on the risk and benefits of anticoagulants in this challenging population and comparisons between warfarin and DOACs, and review future directions including options for non-pharmacologic stroke prevention.

## 1. Introduction

Among patients with end-stage renal disease (ESRD), cardiovascular disease is a major driver of morbidity and mortality. Atrial fibrillation (AF) and ESRD have many shared risk factors including age, hypertension, diabetes mellitus, and vascular disease. As such, the ESRD population appears to have a special predilection towards developing AF. Observational data indicate that AF prevalence may be as high as 25% in closely monitored ESRD patients on chronic intermittent hemodialysis (IHD) [1,2]. The prevalence has also increased over the last two decades likely reflecting heightened awareness, improved monitoring, and increased longevity with dialysis [3].

A diagnosis of AF has been independently associated with increased risk of thromboembolism, hospitalization, and death in this already highly co-morbid population [3,4,5,6,7]. In addition to the association with traditional cardiovascular risk factors, AF establishes a hypercoagulable state through excess endothelial dysfunction, platelet aggregation, and impaired blood flow in the left atrium [8,9,10]. The annual incidence of stroke in ESRD patients with AF has been estimated at approximately 5%—three-fold higher than a matched cohort without documented AF [4]. Furthermore, stroke in ESRD portends a very poor prognosis with 1 in 3 being a fatal event and a majority resulting in death within 1 year [11]. Widely used risk prediction models like the CHA2DS2-VASc score have also shown promise in predicting thromboembolic events in ESRD [12,13]. Yet, AF has been associated with a 5-fold higher risk of stroke in ESRD patients even in those with a CHA2DS2-VASc of 0 when compared with patients with normal renal function (4.2 vs. 0.8 per 100 person-years) [13]. In ESRD patients with AF with a CHA2DS2-VASc of at least 2 the risk can exceed 7 per 100 person-years [13]. Disturbingly, one year mortality with an AF diagnosis has been shown to exceed 1 in 3—double the rate seen in ESRD without AF [3].

Despite ongoing efforts to address these alarming outcomes, the best approach for stroke prevention in AF in the setting of ESRD remains controversial. Although excess thrombotic events have been clearly established in renal disease, there is also substantial concern for excess bleeding related to uremic platelet dysfunction, routine use of systemic heparin during IHD, and an increased need for invasive procedures. As a result, risk of major bleeding at baseline is twice as high in patients with renal disease as those with normal renal function and nearly three-fold higher in those who require dialysis [13,14,15,16]. The desire to provide a net benefit in the face of a delicate balance of both increased bleeding and thrombotic risk sets the ESRD AF population apart from the average AF population with regards to the use of systemic anticoagulation.

## 2. Anticoagulation in Atrial Fibrillation with End-Stage Renal Disease

### 2.1. Warfarin

Until about 10 years ago, warfarin was the only available oral anticoagulant for stroke prevention in AF. Yet, the prospective randomized controlled trials assessing the efficacy of warfarin in stroke prevention excluded patients with severe renal disease defined as an estimated glomerular filtration rate (eGFR) < 30 mL/min [17]. Although warfarin is not contraindicated, pharmacokinetic considerations unique to this population should be taken into account. Warfarin is extensively metabolized by the Cytochrome P450 (CYP) system of enzymes in the liver and primarily by CYP2C9 [18]. Evidence exists to suggest that CYP2C9 is downregulated in patients with ESRD, leading to impaired non-renal clearance and bioavailability of warfarin as well as other drugs [19]. As a result, drug interactions with warfarin also become exaggerated. Warfarin anticoagulation in severe renal dysfunction is associated with a requirement for lower warfarin doses and, ultimately, has been linked to worse overall control when compared to patients with normal renal function [20].

When compared to the general population, meta-analysis has shown that ESRD patients on warfarin have a 10-fold higher risk of bleeding and up to a two-fold higher risk of bleeding compared to ESRD patients not on anticoagulation [16,21,22]. Of particular concern, warfarin use appears to increase intracranial hemorrhage risk in ESRD AF patients [22]. In one study, the risk of hemorrhagic stroke doubled without any significant difference in gastrointestinal hemorrhage [23]. Many of these bleeding events occur when prothrombin time is greater than the target range; suggesting that labile International Normalized Ratio (INR) is at least partially to blame [20,24]. In the face of this significant increase in bleeding, several large retrospective studies showed either no benefit in stroke prevention [14] and, in some studies, warfarin was associated with an increase in risk of not only hemorrhagic but also ischemic stroke [25,26,27]. Some investigators have hypothesized that the increased stroke risk may be a reflection of documented acceleration in vascular and valvular calcification as a result of warfarin use in AF ESRD patients [28,29,30]. Substantial concerns were raised regarding the risk-to-benefit ratio of warfarin anticoagulation in the ESRD AF population and this led to some guidelines recommending against routine anticoagulation in this group [31].

Evidence for a potential benefit of warfarin came from two more recent registry studies. A large retrospective analysis of Danish national registries included a subgroup of 1728 patients with ESRD AF on dialysis and, in those with a CHA2DS2-VASc of at least 2, warfarin use was associated with a 15% reduction in all-cause mortality over a median follow-up of about 3 years. This study also showed trends in the reduction of cardiovascular mortality and a composite end-point of bleeding, stroke, and thromboembolism with warfarin use but these did not reach statistical significance [13]. Another retrospective study utilizing the US Renal Data System identified 1838 patients with ESRD who were initiated on warfarin within 30 days of a new AF diagnosis. Over a relatively short follow-up of 1 year using intention-to-treat analysis, warfarin initiation demonstrated a 32% reduction in ischemic stroke and a 16% reduction in mortality despite 70% discontinuing the drug during follow-up [32].

**Figure 1:**
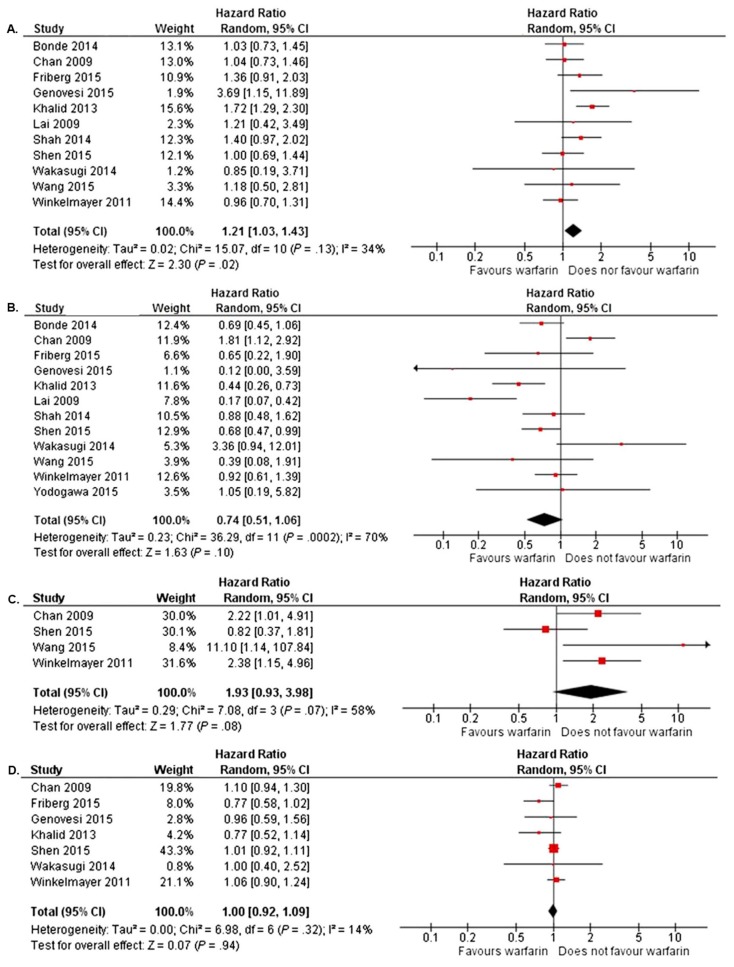
Meta-analysis in patients with end-stage renal disease and atrial fibrillation demonstrating, by forest plots, the risk of four different outcomes associated with warfarin use as compared to no therapy. (**A**) Risk of ischemic stroke. (**B**) Risk of major bleeding. (**C**) Risk of intracranial bleeding. (**D**) Risk of mortality. Reproduced with permission from Van Der Meersch, H.; De Bacquer, D.; De Vriese, A.S., American Heart Journal; published by Elsevier, 2017 [36].

Despite a few positive studies, in multiple meta-analyses, warfarin anticoagulation has failed to demonstrate a consistent reduction in mortality or ischemic stroke in the setting of substantial heterogeneity between individual trials [21,22,33,34,35,36]. Nevertheless, excess bleeding including hemorrhagic stroke has been clearly demonstrated in association with warfarin anticoagulation by many of these pooled analyses [21,22,33,35]. The results of the most recent of these meta-analyses are shown in Figure 1. In this study, a trend towards reduction in ischemic stroke was noted, which did not reach statistical significance, but also a significant increase in bleeding when compared to no therapy. There was no difference in mortality between groups and intracranial bleeding risk nearly doubled. The authors cautioned that the stroke prevention may have been underestimated because many trials included substantial proportions of patients with low stroke risk (CHA2DS2-VASc < 2) [36].

Warfarin anticoagulation has been shown to be ineffective and potentially harmful in maintaining vascular graft patency in patients undergoing IHD [37]. Similarly, dialyzer clotting does not appear to be inhibited by therapeutic warfarin anticoagulation [38]. Although it has not been directly studied, a requirement for the use of heparin for line patency during IHD likely further increases bleeding risk. This increase in risk could potentially be obviated in patients undergoing peritoneal dialysis (PD). PD patients have typically comprised a small proportion of ESRD observational studies and few studies specifically address this population. Of the patients in the previously mentioned Danish study, 25% received PD. In this study, low stroke risk patients on warfarin (CHA2DS2-VASc of 0) had a higher risk of reaching a composite end-point of thromboembolism and bleeding with PD than with IHD but no difference could be demonstrated between the two dialysis modalities in high-risk patients [13]. Another study of 271 Chinese patients on PD showed more than an 80% reduction in ischemic stroke with warfarin compared to placebo and aspirin, and no difference in intracranial hemorrhage [39].

Another rare but disastrous and life-threatening complication of ESRD that has been strongly associated with warfarin use is calciphylaxis, a condition characterized by metastatic calcific occlusion of microvasculature supplying superficial adipose tissue and skin. The incidence of this condition is estimated to be about 1 in 300 per year in ESRD patients [40] and about half of these have a history of warfarin use [41]. Warfarin use has also been associated with increased mortality in those diagnosed with calciphylaxis [41]. Even outside of the context of ESRD, warfarin use has also been associated with accelerated progression of renal disease, particularly in patients who have been excessively anticoagulated [42,43]. In a large retrospective US administrative database analysis, progression of renal disease was seen more frequently in association with warfarin than with rivaroxaban or dabigatran [44]. The potential for warfarin to accelerate the decline in renal function becomes especially relevant in patients with solitary kidney, renal transplant recipients, and those with ESRD who have not yet reached a requirement for dialysis and those who are still producing urine. There are several hypothesized mechanisms by which warfarin exacerbates both calciphylaxis and renal disease progression, but the precise pathophysiology remains unclear [30,44].

The above controversies have led to substantial variability in guideline recommendations. The 2014 US guidelines for AF management and the 2019 focused update give class IIB recommendations for warfarin anticoagulation in ESRD patients with AF who have an elevated stroke risk (CHA2DS2-VASc of at least 2) [45,46]. This is in contrast to the 2012 Canadian AF guidelines who recommend not routinely giving anticoagulation to patients with ESRD AF and the 2016 European guidelines which did not make any strong statement based on a lack of evidence [31,47]. No major guidelines make any special mention with regards to PD.

Other challenging populations are those with ESRD and AF, along with either recent coronary artery stenting or recently implanted bioprosthetic valves, including transcatheter aortic valves. No high-quality evidence exists to guide management in these populations. However, with the significantly increased bleeding risk associated with prerequisite antiplatelet therapy, we believe that the addition of anticoagulation should be reserved only for those at the highest risk of thromboembolism. Besides, the recently completed GALILEO trial of routine rivaroxaban 10 mg versus antiplatelet therapy after TAVR demonstrated a higher risk of death or thromboembolic complications, and a higher risk of bleeding in the rivaroxaban arm [48]. Patients with AF or severe renal impairment were not included in that trial, but the bleeding risk should be anticipated to be even higher in ESRD patients.

### 2.2. Apixaban

Patients with ESRD have thus far been excluded from all published prospective clinical trials assessing direct oral anticoagulants (DOACs) in AF. Apixaban became an attractive alternative to warfarin after the Apixaban for Reduction in Stroke and Other Thromboembolic Events in Atrial Fibrillation (ARISTOTLE) trial demonstrated superiority for stroke prevention, less major bleeding, and lower mortality when compared to warfarin in patients without severe kidney disease [49]. Apixaban is a direct factor Xa inhibitor which is primarily cleared through biliary and direct intestinal excretion with the lowest renal elimination of all DOACs (27%) and very little clearance by IHD [50,51,52]. Small pharmacokinetic studies in ESRD patients showed that single doses result in only modest increases in plasma levels when compared to those with normal renal function [51,53].

Despite an initial absence of data on clinical outcomes, the Federal Drug Administration (FDA) approved the use of apixaban in ESRD patients at the standard dose of 5 mg twice daily with dose reduction to 2.5 mg daily only in patients 80 years of age or older and those who weigh 60 kg or below [54]. The importance of appropriate dose reductions for all DOACs was highlighted in a large US administrative database of all comers (the proportion of patients with ESRD was not specified in this study). That analysis demonstrated that patients who did not follow the labeled dose reduction parameters suffered excess bleeding and, in patients where the dose was inappropriately reduced, an increase in stroke risk was noted. [55]. However, one study has suggested that the 5 mg twice daily dosing regimen for apixaban in ESRD patients may result in supratherapeutic drug levels once steady state is reached following multiple doses [56].

Pharmacokinetics aside, whether clinical outcomes and adverse effects in ESRD patients with AF are comparable to those seen in the ARISTOTLE trial remained unclear at the time of FDA approval of apixaban. The use of various DOACs in clinical practice, occasionally off-label, has led to several retrospective observational studies assessing the safety and efficacy of these drugs in the ESRD AF population (Table 1) [57,58,59,60,61,62]. For apixaban specifically, three small single center analyses proposed that outcomes of thromboembolism and mortality with apixaban were comparable to those seen with warfarin with perhaps fewer bleeding events [58,59,60]. A subsequent large analysis of over 25,000 patients in the US Renal Data system found an overall reduction of 28% in major bleeding with similar rates of thromboembolic events and mortality when comparing apixaban to a matched cohort of ESRD AF patients on warfarin [61]. Additional sensitivity analysis showed that the 5 mg twice daily dose was associated with significant reductions in thromboembolic events and death when compared to both warfarin as well as reduced-dose apixaban (2.5 mg twice daily). There were no differences in bleeding between the two apixaban doses. Interestingly, similar to some of the warfarin studies mentioned above, more than two-thirds of patients discontinued oral anticoagulation within 12 months in both the apixaban and warfarin groups equally [61]. In addition, despite the favorable associations noted for apixaban over warfarin for some of the endpoints, it should be noted that bleeding rates, particularly intracranial, were very high with apixaban in this real-world ESRD population. While thought-provoking, the observational nature of these data with a potential for residual confounding is a notable limitation.

Given the mounting data to support efficacy in stroke prevention and safety, the 2019 focused update of the US Atrial Fibrillation Management Guidelines added the use of apixaban as an alternative to warfarin with a class IIB recommendation for anticoagulation in ESRD AF on dialysis with elevated stroke risk [46]. The aforementioned studies involving outcomes with apixaban were also published after the 2014 US guidelines, 2012 Canadian guidelines, and 2016 European guidelines and, as such, it is not surprising that none of these have made statements regarding the use of this drug in ESRD [31,45,47]. Apixaban has shown promise when compared to warfarin and may in fact be superior with regards to bleeding events in this vulnerable population. Randomized data is needed to validate these findings. Furthermore, very few ESRD AF patients on PD have been included in the analyses comparing apixaban to warfarin. In this population, it remains unclear to what extent the demonstrated benefits of stroke prevention without an increase in intracranial hemorrhage seen with warfarin also apply to apixaban [39].

### 2.3. Other Oral Anticoagulants

Although three other DOACs are available in addition to apixaban, the pharmacokinetics of these medications in chronic kidney disease vary widely. Dabigatran has the highest degree of renal elimination (80%), followed by edoxaban (50%), and then rivaroxaban (33%) [52]. Out of these agents, IHD results in meaningful clearance (~65%) only for dabigatran [63]. There are no prospective trials which have assessed the safety and efficacy of these drugs in ESRD AF and, in contrast to apixaban, very few observational studies have been published (Table 1).

Like apixaban, it stands to reason that rivaroxaban could be anticipated to be more suitable for use in ESRD AF given its low renal elimination. A dose-finding study reported similar steady-state plasma concentrations using 10 mg daily doses in ESRD when compared to a 20 mg daily dose in controls with normal renal function [64]. Two observational studies have focused on rivaroxaban in ESRD with rather conflicting results regarding its effect on bleeding. The first was an analysis of a North American dialysis database which included 240 patients on rivaroxaban (two-thirds on 15 mg daily and one-third on 20 mg daily). This showed higher major and non-major bleeding risks with a 40% increase in hospitalization or death from bleeding when compared to warfarin [57]. However, a more recent study utilizing a US insurance base which identified 1900 patients where 90% had stage 5 chronic kidney disease and/or required IHD, showed a 30% reduction relative to warfarin in major bleeding with only 40% taking rivaroxaban doses less than 20 mg daily [62]. The reason for the discrepancy in bleeding outcomes between these two studies remains unclear. Since the more recent study did not specify the exact proportion of patients on IHD, it is possible that overall renal function was better in this population resulting in improved outcomes despite a larger proportion of patients taking full dose rivaroxaban.

Having the highest renal elimination among all DOACs, dabigatran has generally been regarded as a poor choice in ESRD. Despite evidence of clearance by IHD, dabigatran has been shown to accumulate in ESRD patients at levels twice as high as those seen in controls with normal renal function [63]. One observational study assessed outcomes in 280 patients with ESRD and off-label use of the drug early after the drug was first approved in the US. This study reported an 80% increase in hemorrhagic death and a 50% increase in death or hospitalization from bleeding when compared to warfarin [57]. Another retrospective database analysis showed nearly a 4-fold increase in major bleeding (primarily driven by gastrointestinal bleeding) with dabigatran as compared to warfarin in patients with severe kidney disease (eGFR < 30 mL/min) [65].

Edoxaban was the last of the DOACs to obtain approval by the FDA. With the exception of a study showing poor clearance with IHD after a single dose of edoxaban, little data exists regarding its pharmacokinetics, safety, or efficacy in ESRD AF [66].

Based on a paucity of evidence, the 2019 focused update of the US atrial fibrillation management guideline recommends against the use of rivaroxaban, dabigatran, and edoxaban in ESRD AF [46]. The 2012 Canadian guidelines and the 2016 European guidelines do not make any statements for or against the use of these agents in ESRD patients [31,47].

### 2.4. Future Directions

As highlighted above, there is a need for randomized prospective data for the use of systemic anticoagulation in ESRD patients with AF who have an elevated stroke risk. The pharmacokinetics and promising observational data suggesting reduction in bleeding when compared to warfarin, makes apixaban the most attractive of the DOACs for a large scale ESRD trial. The ideal analysis would include three groups to address not only comparative safety and efficacy between warfarin and a DOAC (experimental groups) but also whether systemic anticoagulation benefits this population as opposed to placebo (control group). A subgroup analysis or, even better, a separate trial should also focus on patients undergoing PD since this group has many characteristics that are distinct from the IHD population. Furthermore, such a study will likely require multicenter, international collaboration in order to achieve adequate power for detecting differences in thromboembolic, bleeding, and mortality outcomes.

Randomized controlled trials are underway comparing apixaban with warfarin in dialysis patients (Table 2). The AXADIA study (Compare Apixaban and Vitamin-K Antagonists in Patients with Atrial Fibrillation and End-Stage Kidney Disease) is randomizing patients to apixaban 2.5 mg twice a day or phenprocoumon—a vitamin K antagonist approved for use in some European countries. This study is currently recruiting with an estimated completion date of July 2022 (NCT02933697) [67]. The RENAL-AF trial (RENal Hemodialysis Patients ALlocated Apixaban Versus Warfarin in Atrial Fibrillation) randomized patients to either apixaban 5 mg twice a day or warfarin. Selected patients received low-dose 2.5 mg twice daily apixaban (NCT02942407). After enrolling 154 patients, RENAL-AF was stopped early and short of the 762 patient target due to slow enrollment and finite resources. The results were recently presented at the 2019 Scientific Sessions of the American Heart Association but have not been published in a peer-reviewed journal as of yet. Similar rates of major and clinically-relevant non-major bleeding were reported between apixaban and warfarin, but the investigators admitted that power was limited due to small sample size and low event rate [68]. Of note, neither of these trials include a control group. Whether the risk-to-benefit ratio favors systemic anticoagulation as opposed to no therapy in the ESRD AF population has yet to be demonstrated.

## 3. Non-Pharmacological Stroke Prophylaxis

Patients with ESRD have an increased risk of bleeding as discussed above, and systemic anticoagulation may not be the ideal approach for stroke prevention in ESRD AF patients regardless of the agent used, especially in those with a history of major bleeding. As such, non-pharmacological measures for stroke prophylaxis become highly relevant in this population.

The left atrial appendage (LAA) has been heavily implicated in the pathogenesis of cardioembolic stroke in AF as about 90% of thrombi in patients with AF are formed in the LAA [69]. LAA isolation has gained traction in clinical practice in patients who are at an elevated stroke risk but also are poor candidates for long-term anticoagulation. Traditionally, the LAA has been targeted via clipping, suture ligation, or amputation with an open or minimally-invasive approach during cardiac surgery. However, ESRD patients are also at an exceedingly high surgical risk and these approaches are typically reserved for patients undergoing cardiac surgery for another indication [46].

Percutaneous LAA occlusion using the WATCHMAN device (Boston Scientific, Marlborough, MA) demonstrated non-inferiority compared to warfarin with regards to combined ischemic and hemorrhagic stroke prevention in two randomized trials [69,70]. A subgroup analysis of one of these trials showed that relative risk reductions in both stroke and bleeding were highest in patients with an eGFR of less than 60 mL/min [71]. Two retrospective single center analyses have also reported greater than expected reductions in both bleeding and thromboembolism in patients with chronic kidney disease following WATCHMAN implantation. Both studies only had a small number of patients with severe renal disease and neither reported whether any were on dialysis [72,73].

The Amplatzer Cardiac Plug (ACP; St. Jude Medical, Minneapolis, MN) is another device designed for percutaneous LAA occlusion and used primarily in Europe. A multicenter registry of 375 patients with chronic kidney disease (including 14 patients on dialysis) showed no difference in major periprocedural complications and similar reductions in stroke and bleeding risk when compared to those with normal renal function [74].

Current guidelines do not specifically address LAA occlusion or non-pharmacologic stroke prevention in ESRD AF [31,46,47]. The WATCH-HD (WATCHMAN Device in Patients With Non-valvular Atrial Fibrillation and End-stage Chronic Kidney Disease on Hemodialysis) trial is currently recruiting and will randomize ESRD AF patients with elevated stroke and bleeding risk to either WATCHMAN implantation or no intervention (NCT03446794).

## 4. Conclusions

The ESRD AF population carries substantially increased risks of both thromboembolism and major bleeding at baseline. The risk-to-benefit ratio with systemic anticoagulation in this patient population, therefore, is unlikely to mirror that of the general AF population and this uncertainly needs to be considered when formulating an approach for stroke prevention. Warfarin has several potential disadvantages unique to ESRD AF patients and observational data suggests that apixaban may be associated with an acceptable safety profile. Results of ongoing randomized trials are eagerly awaited to further clarify these observations. A non-pharmacological approach such as percutaneous LAA occlusion could theoretically be particularly attractive in this high bleeding risk population, but data are currently lacking. The best approach for stroke prevention in this highly complex and co-morbid population requires further assessment in prospective studies.

## Figures and Tables

**Table 1:** Summary of retrospective studies comparing the efficacy and safety of direct oral anticoagulants versus warfarin in end-stage renal disease patients with atrial fibrillation.

Study	Chan et al. (2015) [57]	Stanton et al. (2017) [58]	Sarrat et al. (2017) [59]	Reed et al. (2018) [60]	Siontis et al. (2018) [61]	Coleman et al. (2019) [62]
Sample size	29,977	357 (146 after matching)	160	124	25,523 (9404 after matching)	6744
Anticoagulation at baseline (%)	244/29,977 (0.8)—Rivaroxaban 281/29,977 (0.9)—Dabigatran 8064/29,977 (27)—Warfarin	73/146 (50)—Apixaban 73/146 (50)—Warfarin	40/160 (25)—Apixaban 120/160 (75)—Warfarin	74/124 (60)—Apixaban 50/124 (40)—Warfarin	2351/9404 (25)—Apixaban 7053/9404 (25)—Warfarin	1896/6744 (28)—Rivaroxaban 4848/6744 (72)—Warfarin
DOAC dose (%)	165/244 (68)—Rivaroxaban 15 mg OD 79/244 (32)—Rivaroxaban 20 mg OD 238/281 (85)—Dabigatran 75 mg BID 43/281 (15)—Dabigatran 150 mg BID	45/73 (62)—Apixaban 2.5 mg BID 27/73 (37)—Apixaban 5 mg BID 1/73 (1)—Apixaban 10 mg BID	23/40 (58)—Apixaban 2.5 mg BID 17/40 (43)—Apixaban 5 mg BID	15/74 (20)—Apixaban 2.5 mg BID 59/74 (80)—Apixaban 5 mg BID	1317/2351 (56)—Apixaban 2.5 mg BID 1034/2351 (44)—Apixaban 5 mg BID	734/1896 (39)—Rivaroxaban <20 mg OD 1162/1896 (61)—Rivaroxaban 20 mg OD
Age, years (mean ± SD or (range))	**67 ± 12—Rivaroxaban** **68 ± 12—Dabigatran** **71 ± 11—Warfarin**	79 ± 12—Apixaban 79 ± 14—Warfarin	71 (60–81)—Apixaban 67 (53–80)—Warfarin	60 ± 15—Apixaban 62 ± 14—Warfarin	69 ± 11—Apixaban 68 ± 12—Warfarin	72 (63–80)—Rivaroxaban 72 (63–80)—Warfarin
Female (%)	96/244 (39)—Rivaroxaban 155/281 (41)—Dabigatran 3129/8064 (39)—Warfarin	44/73 (60)—Apixaban 43/73 (59)—Warfarin	20/40 (50)—Apixaban 62/120 (52)—Warfarin	36/74 (49)—Apixaban 19/50 (38)—Warfarin	1071/2351 (46)—Apixaban 3257/7053 (47)—Warfarin	789/1896 (42)—Rivaroxaban 1862/4848 (38)—Warfarin
CHA2DS2-VASc (mean ± SD or (range))	2 ± 1—Rivaroxaban 2 ± 1—Dabigatran 2 ± 1—Warfarin	6 ± 1—Apixaban 6 ± 2 - Warfarin	5 (1–6)—Apixaban 5 (2–7)—Warfarin	4 ± 1—Apixaban 4 ± 1—Warfarin	4 ± 1—Apixaban 4 ± 1—Warfarin	4 (2–5)—Overall cohort
Atrial fibrillation at baseline (%)	29,977/29,977 (100)	53/73 (73)—Apixaban 53/73 (73)—Warfarin	32/40 (80)—Apixaban 81/120 (68)—Warfarin	**29/74 (39)—Apixaban** **29/50 (58)—Warfarin**	9404/9404 (100)	6744/6744 (100)
Dialysis at baseline (%)	29,977/29,977 (100)	20/73 (27)—Apixaban 20/73 (27)—Warfarin	160/160 (100)	124/124 (100)	9,404/9,404 (100)	~5930/6744 (88)—Overall cohort (stage 5 CKD and/or HD)
Cohort matching at baseline	-None -Co-variate adjusted Poisson regression for HR analysis	-Renal function -Anticoagulation indication	-None	-None	-Prognostic score for “death”	-Baseline co-variates (propensity scores)
Mean follow-up, months	4—Rivaroxaban 5—Dabigatran 6—Warfarin	12—Apixaban 18—Warfarin	NA	10	~3—Apixaban ~5—Warfarin	17
Major bleeding events, per 100 person-years	68—Rivaroxaban 83—Dabigatran 36—Warfarin	9—Apixaban 12—Warfarin	0—Apixaban 7/120 (6%)—Warfarin (follow-up length NA)	7—Apixaban 24—Warfarin	20—Apixaban 23—Warfarin	4—Rivaroxaban 6—Warfarin
Major bleeding vs. warfarin (HR (95% CI))	**1.38 (1.03–1.83)—Rivaroxaban** **1.48 (1.21–1.81)—Dabigatran**	0.49 (0.18–1.31)	0.19 (0.01–3.35)	**0.15 (0.05–0.46)** ^¶^	**0.72 (0.59–0.87)**	**0.68 (0.47–0.99)**
Non-major bleeding events, per 100 person-years	149—Rivaroxaban 121—Dabigatran ~4223/8064 (52)- Warfarin	11—Apixaban 13—Warfarin	5/40 (13%)—Apixaban 7/120 (6%)—Warfarin (follow-up length NA)	24—Apixaban 23—Warfarin	NA	NA
Non-major bleeding vs. warfarin (HR (95% CI))	**1.35 (1.11–1.65)—Rivaroxaban** 1.17 (1.00–1.38)—Dabigatran	1.37 (0.45–4.18)	2.31 (0.69–7.72)	NA	NA	NA
Thromboembolic events, per 100 person-years	11—Rivaroxaban 11—Dabigatran 6—Warfarin	8—Apixaban ^‡^ 12—Warfarin ^‡^	NA	0—Apixaban 0—Warfarin	12—Apixaban 12—Warfarin	1—Rivaroxaban2—Warfarin
Thromboembolism vs. warfarin (HR (95% CI))	NA *	1.0 (0.23–4.23) ^‡^	NA	NA	0.88 (0.69–1.12)	0.55 (0.27–1.10)
Mortality events, per 100 person-years	16—Rivaroxaban ^†^ 19—Dabigatran ^†^ 10—Warfarin ^†^	NA	NA	NA	24—Apixaban 25—Warfarin	NA
Mortality vs. warfarin (HR (95% CI))	1.71 (0.93–3.12)—Rivaroxaban ^†^ **1.78 (1.18–2.68)—Dabigatran** ^†^	NA	NA	NA	0.85 (0.71–1.01)	NA

Organized in order of date published. Bold text indicates statistically significant differences between groups with a *p*-value less than 0.05. BID = twice-daily; CI = confidence interval; CKD = chronic kidney disease; DOAC = direct oral anticoagulant; HD = hemodialysis; HR = hazard ratio; NA = not available; OD = once daily; SD = standard deviation. * Too few events to draw meaningful conclusion ^†^ Only reported for deaths related to hemorrhage. ^‡^ Only reported in patients with atrial fibrillation at baseline. ^¶^ Only reported for overall bleeding without differentiation between major and non-major.

**Table 2:** Summary of prospective randomized trials evaluating the efficacy and safety of apixaban versus warfarin in end-stage renal disease patients with atrial fibrillation.

Trial	Methods	Inclusion Criteria	Primary Outcomes	Secondary Outcomes	Enrollment	Expected Completion
RENAL-AF (NCT02942407)	Open-label randomization to apixaban (5/2.5 mg) versus warfarin (INR 2–3) for up to 15 months	-18 years or older -AF with CHA2DS2-VASc ≥ 2 -ESRD on HD > 3 months -OAC candidate	-Time to first major or clinically relevant non-major bleeding event	-Stroke or systemic embolism -Mortality -Apixaban phramacokinetics	US, Multicenter (762 patient target)	August 2019 (154 patients enrolled at completion)
AXADIA (NCT02933697)	Open-label randomization to apixaban (2.5 mg) versus phenprocoumon (INR 2–3) for 6–24 months	-18 years or older -AF with CHA2DS2-VASc ≥ 2 -ESRD on HD > 3 months -OAC candidate	-Time to first major or clinically relevant non-major bleeding event	-Thromboembolism -Apixaban pharmacokinetics (*n* = 28)	Germany, Multicenter (222 patient target)	July 2022 (Recruiting)

AF = atrial fibrillation; ESRD = end-stage renal disease; HD = hemodialysis; INR = International Normalized Ratio; OAC = oral anticoagulation.
